# Cell-mediated matrix deformations and cell–cell adhesions determine epithelial collective cell migration phenotypes

**DOI:** 10.1063/5.0295506

**Published:** 2025-12-17

**Authors:** Corinne E. Leonard, Jessanne Y. Lichtenberg, Hazel R. Sterling, Jesse Rolston, Sydnie K. Tran, Priscilla Y. Hwang

**Affiliations:** 1 Department of Biomedical Engineering, Virginia Commonwealth University, Richmond, Virginia 23284, USA; 2 Massey Comprehensive Cancer Center, Virginia Commonwealth University, Richmond, Virginia 23298, USA

## Abstract

Successful development of tissue structures requires different collective cell migration patterns or phenotypes. Two examples of collective migration phenotypes in epithelial morphogenic processes, such as tubulogenesis, are rotational and invasive. Rotational collective migration phenotypes (RCM) typically lead to acinar structures, and invasive collective migration (ICM) phenotypes lead to duct-like structures. How cells adopt these different phenotypes is still largely unknown. Here, we investigate how cell–cell adhesion marker P-cadherin (CDH3) and mechanical cell–matrix interactions, including matrix deformations, protrusions, and focal adhesions, control rotational or invasive phenotypes during tubulogenesis. To accomplish our objective, we created a custom 3D microfluidic assay to perform live-cell imaging of epithelial clusters or cysts [wild-type (WT) and CDH3-depleted (CDH3^-/-^)] undergoing tubulogenesis, while simultaneously measuring matrix deformation rates. Our findings reveal WT epithelial cysts maintain rotational phenotypes, but transition to an invasive phenotype to undergo tubulogenesis. Furthermore, we demonstrate ICM phenotypes correlate with higher matrix deformation rates compared to rotational phenotypes. Our studies reveal CDH3 is required for epithelial cysts to transition from rotational to ICM phenotypes, associated with decreased matrix deformation rates. Without CDH3, epithelial cysts lose their ability to adopt ICM phenotypes, which can be rescued by RhoA activation. Finally, we demonstrate that the RhoA-rescued ICM phenotype is mediated, in part, by increased matrix deformation rates and vinculin recruitment to focal adhesion sites.

## I. INTRODUCTION

Tissue morphogenesis requires precise cell migration or motility, which involves the coordination of cell–cell interactions and reciprocal interactions between cells and their extracellular matrix (ECM).^1–6^ Changes in cell migration phenotypes or patterns can determine tissue shape.^2,3,7,8^ For example, branching morphogenesis, or tubulogenesis, is initiated when growth factors stimulate a few individual cells within a cyst (i.e., cluster) to extend protrusions that adhere to the ECM.^2,8,9^ These cells lead cohorts of their neighbors to migrate through the ECM to form branched tubules through a process known as collective migration.^4,9,10^ During collective migration, cells move together as an entire unit in a dynamic fashion, where cells can undergo different cell motility and adhesion patterns.^11,12^ *In vivo*, epithelial cysts must adopt different collective migration phenotypes to execute successful tissue development.^8,13,14^ One collective migration phenotype is known as invasive collective migration (ICM), which describes cell extensions that protrude from a primary cell cluster, typically observed during development of tubules in epithelial tubulogenesis^9,13,15,16^ or in cancer metastasis.^17–19^ It is believed that cells establish mature protrusions and adhesions with the extracellular matrix to drive an ICM phenotype.^8,9,20^ Another collective migration phenotype is known as rotational collective migration (RCM), which describes cells rotating collectively around a center axis and is essential for the development of circular and cylindrical structures, such as acinar mammary glands.^13,20–22^ In RCM, it is believed that cells have strong cell–cell adhesions, which keep them actively rotating and maintaining spherical morphology.^22,23^ Some work suggests that rotational cell movement occurs due to spatial confinement,^23–25^ though another group demonstrated that colon adenocarcinoma epithelial cells can adopt rotational phenotypes spontaneously.^26^

A very active area of research is understanding how cells use mechanical forces to sense and respond to their physical environment.^27–30^ Studies have investigated contributions of forces in dictating cell migration phenotypes.^31–33^ Within a 2D endothelial cell sheet, each individual cell can adopt a different cell migration phenotype (i.e., rotational, translating, or proliferating) that can guide the larger sheet migration.^34^ This study investigated which forces dictate the 2D migration phenotype: rotational cell phenotype is modulated by intercellular forces, including torque, whereas translating cell phenotype is dominated by cell–matrix forces (i.e., traction forces).^34^ During sheet migration of Madin–Darby Canine Kidney (MDCK) epithelial cells, the cells at the front edge send out protrusions, or migrating fingers, characteristic of invasive phenotypes, and traction force measurements demonstrate the migrating fingers exert higher traction forces compared to cells in the back of the sheet.^35^ Similarly, in 2D, invasive migration of single-cell breast cancer cells correlates with increases in traction forces compared to non-metastatic cells.^36^ In 3D, breast cancer cell lines can form clusters and spontaneously adopt RCM and ICM phenotypes; RCM clusters exhibit global matrix remodeling, and ICM clusters use local matrix remodeling techniques.^20^ Another study demonstrated that invasive ductal cells cultured in collagen 1 hydrogels can sense changes in extracellular matrix tensions.^37^ However, how the collective migration phenotype of epithelial cysts undergoing tubulogenesis in 3D correlates with changes in forces or matrix deformation rates is still not fully understood.

In this study, we investigated which phenotypes (ICM or RCM) 3D epithelial cysts adopt during the tubulogenesis process and how that correlates with matrix deformation rates. We developed a novel assay to measure 3D extracellular matrix deformation in microfluidic models using fluorescent microbeads. Using our microfluidic model, we can perform live-cell imaging to observe cysts undergoing tubulogenesis. Simultaneously, we can quantify cell-mediated matrix deformation rates in real time. In prior work, we demonstrated P-cadherin (CDH3), a cell–cell adhesion marker, regulates cell protrusions in epithelial cysts during tubulogenesis.^16,17^ Specifically, without CDH3, epithelial cysts lost their ability to generate mature protrusions that are required for tubulogenesis.^16,17^ Thus, in this study, we compare wild-type (WT) and CDH3-depleted (CDH3^-/-^) epithelial cysts as our models to correlate how cellular protrusions, matrix deformations, cell adhesions, and collective migration phenotypes are interlinked. Our findings demonstrate the majority of epithelial cysts exist in RCM before tubulogenesis and transition to ICM when undergoing tubulogenesis. Furthermore, clusters undergoing ICM exert higher matrix deformation rates on their surrounding ECM compared to clusters undergoing RCM. Epithelial cysts with depleted CDH3 lose their ability to transition to ICM and always exist in RCM, suggesting CDH3 controls the ability for epithelial cysts to change their migration phenotype from RCM to ICM. Finally, our findings demonstrate that RhoA activation rescues the ICM phenotype in CDH3-depleted cysts, mediated by increased 3D matrix deformation rates, 2D traction forces, and number of vinculin focal adhesions.

## II. RESULTS

### A. Epithelial cysts send out protrusions and generate higher matrix deformation rates after HGF stimulation compared to non-stimulated controls

**FIG. 1.**
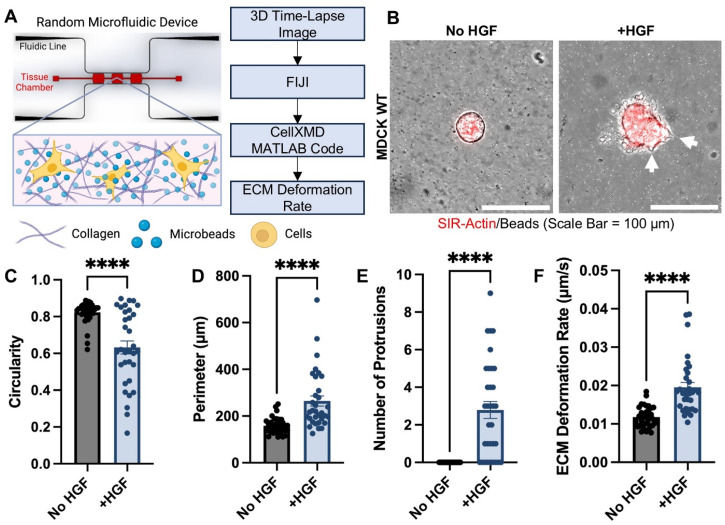
MDCK cysts respond to chemical stimulation via increased invasiveness and ECM deformation upon addition of HGF. (a) Diagram highlighting the experimental setup and workflow of the CellXMD assay within a microfluidic device. (b) Representative images of MDCK wild-type (WT) epithelial cysts, both untreated (No HGF) and treated with HGF (+HGF) (white arrows highlight protrusions). Quantification of cyst morphology: (c) circularity, (d) perimeter, and (e) number of protrusions. (f) ECM deformation quantification of MDCK cysts, untreated (No HGF) and treated with HGF (+HGF). n = 33–36 cysts per condition, from at least three independent trials. Mean ± SEM, ^****^p < 0.0001; Welch's t-test.

We asked whether epithelial cysts change cell–matrix interactions when undergoing tubulogenesis, a form of epithelial morphogenesis. It is well established that epithelial cysts remain round with a hollow lumen before undergoing tubulogenesis. After hepatocyte growth factor (HGF, an established method to induce tubulogenesis^9,15,38^) stimulation, epithelial cysts begin to send out protrusions that eventually develop into tubules.^15,16^ Thus, we use this as our model to understand whether cyst protrusions are associated with changes in cell–matrix interactions, mediated by matrix deformation rates. To measure matrix deformation rates, we encapsulated fluorescent beads and Madin–Darby Canine Kidney (MDCK) epithelial cysts together in collagen 1 hydrogels and loaded the mixture into our microfluidic devices [Figs. 1(a) and 1(b)]. After culturing cysts in microfluidic devices overnight, we added HGF and performed live-cell imaging every 10 min for ten cycles [Fig. 1(b), supplementary material Videos 1 and 2]. We quantified changes in cyst invasion after HGF stimulation through measuring circularity and perimeter [Figs. 1(c) and 1(d)]. After HGF stimulation, epithelial cysts had significantly decreased circularity and significantly larger perimeters compared to non-stimulated controls, suggesting cysts invaded into the surrounding collagen matrix. The decrease in circularity and increase in perimeter correlated with significantly more cell protrusions compared to non-stimulated groups, which did not have any cell protrusions [Fig. 1(e)].

To measure matrix deformation rate, we tracked individual microbead displacements (in micrometers) over time (in seconds) within the matrix surrounding the cyst and averaged to achieve an ECM deformation rate for each cyst, using our CellXMD MATLAB code (supplementary material S1). Findings reveal epithelial cysts have significantly larger ECM deformation rates after HGF stimulation compared to non-stimulated controls [Fig. 1(f)]. It is difficult to make direct, one-to-one comparisons between our calculated matrix deformation rates with published deformation rates because of differences in experimental setup, types of cells investigated, etc. However, we verified our calculated raw bead displacements fall within a similar range of another published study reporting mean maximum bead displacements.^20^ Our calculated deformation rates are also comparable to matrix deformation rates our group published in another study using static hydrogels.^39^ All together, these findings demonstrate increased invasion of epithelial cysts results in increased protrusions that are associated with increased deformations on the surrounding matrix.

We also investigated whether protrusions are linked to matrix deformations in other cell types. Specifically, we measured protrusions and matrix deformations in epithelial-derived breast cancer cell lines and primary breast cancer cell clusters (commonly known as organoids). In our prior publications, we demonstrated aligned matrix fibers induce directional invasion of breast cancer cells.^17,18^ Using this same model system, we cultured breast cancer cells in aligned collagen and measured changes in cell protrusions and matrix deformation rates [supplementary material Fig. 1(a)]. First, we tested single cells: in aligned fiber environments, individual breast cancer cells send out fewer protrusions compared to cells cultured in randomly oriented environments [supplementary material Fig. 1(b)]. However, breast cancer cells generate higher matrix deformation rates per protrusion in aligned environments compared to randomly oriented environments [supplementary material Fig. 1(c)]. We observed similar behavior during collective cell migration for primary breast tumor cell clusters or organoids [supplementary material Figs. 1(d)–1(f)]. Our findings suggest that directional invasion may be linked to increased matrix deformations and demonstrate our CellXMD assay can be applied broadly to study other types of cell migration.

### B. Epithelial cysts transition from a rotating collective migration (RCM) phenotype to an invasive collective migration (ICM) phenotype after HGF stimulation

**FIG. 2.**
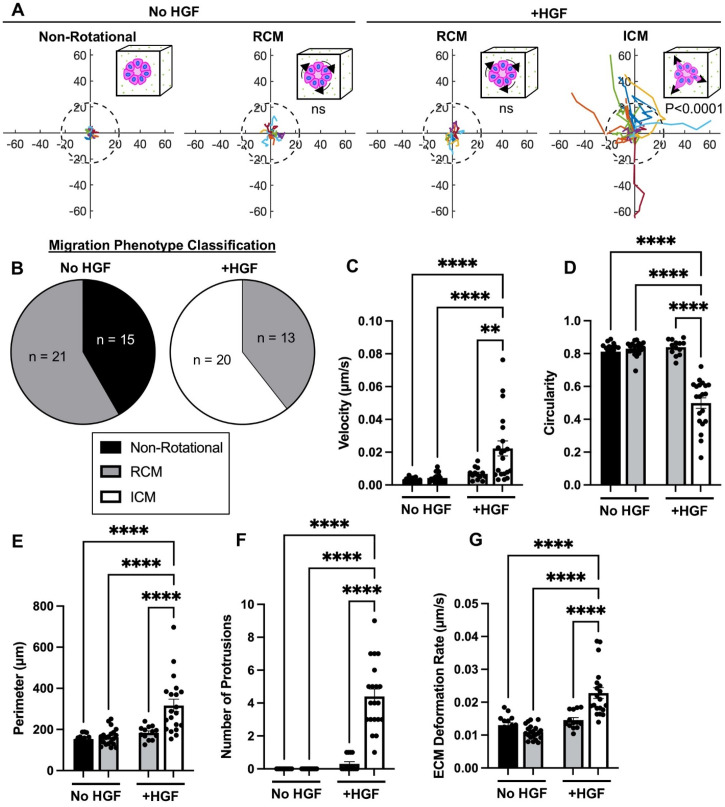
MDCK WT cysts experience varying collective migration phenotypes in 3D collagen gels. (a) Migration maps of WT MDCK cysts depicting total displacement of non-rotational, rotational (RCM), and invasive (ICM) collective migration phenotypes (the dashed circle represents the average cyst area of untreated conditions (No HGF); the axis scale is in micrometers; p-values are relative to the non-rotational phenotype). (b) Classification of migration phenotypes, (c) calculated velocity, (d) circularity, (e) perimeter, (f) number of protrusions, and (g) extracellular matrix deformation of WT MDCK cysts, untreated (No HGF) and treated with HGF (+HGF) [non-rotational (black), RCM (gray), and ICM (white)]. n = 33–36 cysts per condition, from at least three independent trials. Mean ± SEM, ns = not significant, ^**^p < 0.01, ^****^p < 0.0001; ANOVA with Tukey's multiple comparisons test.

Different collective migration phenotypes have been observed throughout development *in vivo*. In the literature, RCM is associated with maintenance of acinar structure, and ICM is associated with ductal structures.^20^ For example, mammary breast epithelial clusters adopt RCM during acinar development to form spherical structures while establishing an endogenous basement membrane,^2,20,40^ whereas clusters adopt ICM in order to form tube structures such as ducts.^20^ *In vitro* studies demonstrated that MDCK epithelial cysts can adopt a RCM phenotype in Matrigel, suggesting similarities in epithelial morphogenesis across mammary and kidney structures.^22^ Earlier we observed a link between cell protrusions and matrix deformation rates in response to HGF stimulation (Fig. 1); thus, we hypothesize epithelial cysts adopt a RCM phenotype before HGF stimulation and transition to an ICM phenotype after HGF stimulation. To test our hypothesis, we characterized collective migration phenotypes before and after HGF stimulation. Before HGF stimulation, epithelial cysts either remained in place without any movement (non-rotational) [supplementary material Video 3] or adopted a RCM phenotype [Figs. 2(a) and 2(b), supplementary material Video 1]. After the addition of HGF, around 60% of cysts transitioned to an ICM phenotype (supplementary material Video 2), and 30% of cysts adopted or maintained a RCM phenotype [Figs. 2(a) and 2(b)]. After separating cysts based on migration phenotype (non-rotational, RCM, and ICM), we quantified differences in migration speed and cyst morphology. Before HGF stimulation, no differences in average velocity were quantified between non-rotational and RCM cysts [Fig. 2(c)]. After HGF stimulation, cysts undergoing ICM had significantly higher average velocity compared to RCM and non-rotational cysts regardless of HGF stimulation [Fig. 2(c)]. Similarly, there were no differences in circularity or perimeter between non-rotational and RCM cysts; but cysts undergoing ICM had significantly decreased circularity and a significantly larger perimeter compared to non-rotational and RCM cysts [Figs. 2(d) and 2(e)]. The decreased circularity and larger perimeter observed in ICM cysts indicate irregularly shaped cysts, indicative of invasive behavior. HGF induces tubulogenesis, and the first step in tubulogenesis is establishing cell protrusions.^9,15,16,38^ In our study, only the cysts with the ICM phenotype sent out multiple protrusions, indicating cysts that have ICM are HGF-responsive [Fig. 2(f)]. The protrusions directly correlated with increased ECM deformation rates, demonstrating cysts interact with the surrounding matrix through mechanical matrix sensing [Fig. 2(g)].

### C. Depletion of CDH3 prevents epithelial cysts from adopting the ICM phenotype, with an associated decrease in cell protrusions and matrix deformation rate

**FIG. 3.**
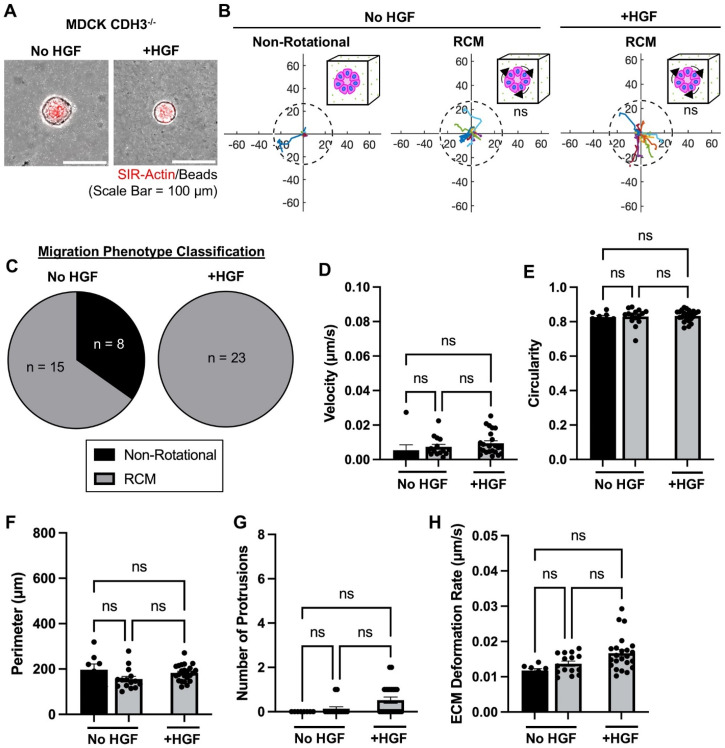
CDH3 is required for the invasive collective migration phenotype upon HGF stimulation. (a) Representative images of CDH3^-/-^ MDCK cysts, both untreated (No HGF) and treated with HGF (+HGF). (b) Migration maps of CDH3^-/-^ MDCK cysts depicting total displacement of non-rotational and rotational (RCM) collective migration phenotypes (the dashed circle represents the average cyst area of untreated conditions (No HGF); the axis scale is in micrometers; p-values are relative to the non-rotational phenotype). (c) Classification of migration phenotypes, (d) calculated velocity, (e) circularity, (f) perimeter, (g) number of protrusions, and (h) extracellular matrix deformation of WT MDCK cysts, untreated (No HGF) and treated with HGF (+HGF) [non-rotational (black) and RCM (gray)]. n = 23 cysts per condition, from at least three independent trials. Mean ± SEM, ns = not significant; ANOVA with Tukey's multiple comparisons test.

We next investigated how the cell–cell adhesion marker, CDH3, regulates the collective migration phenotype and matrix deformations. In our previously published work, we demonstrated an essential role of CDH3 in regulating cell protrusions that are necessary for tubulogenesis.^16,17^ Thus, we asked if CDH3 regulates matrix deformations associated with cell protrusions and ICM phenotypes. We performed live-cell imaging of MDCK CDH3-depleted (CDH3^-/-^) epithelial cysts and assessed migration phenotype and matrix deformation before and after HGF stimulation. Before the addition of HGF, CDH3^-/-^ epithelial cysts adopted a non-rotational (supplementary material Video 4; 35%) or RCM phenotype [Figs. 3(a)–3(c); 65%], like WT cysts (Fig. 2). After HGF stimulation, all CDH3^-/-^ cysts adopted a RCM phenotype [Figs. 3(a)–3(c), supplementary material Video 5]. CDH3^-/-^ cysts did not have any differences in velocity regardless of culture condition [Fig. 3(d)]. We also did not observe any differences in circularity, perimeter, or protrusions [Figs. 3(e)–3(g)]. Finally, CDH3^-/-^ cysts exhibited non-significant changes in ECM deformation rates across non-rotational and RCM groups, regardless of HGF stimulation [Fig. 3(h)]. These findings support previous literature identifying CDH3 as necessary for ICM phenotype epithelial cyst invasion,^16,17^ which is associated with protrusions and ECM deformation rates.

We further performed CDH3 shRNA knockdown (shCDH3-1 and shCDH3-2, compared to shGFP-scramble control) in MDCK cells to confirm findings are specific to CDH3 and not off-target effects associated with CDH3 depletion. Successful knockdown was validated via western blot [supplementary material Fig. 2(a)]. We performed a 2D sheet migration assay to confirm both clones exhibited decreased collective migration potential, as seen in MDCK CDH3^-/-^ cells.^16^ Quantification of fold change in gap closure area compared to time 0 revealed both shCDH3 clones closed the gap more slowly over time compared to the shGFP-scramble control [supplementary material Fig. 2(b)]. MDCK shCDH3-1, shCDH3-2, and shGFP-scramble control cysts were then cultured in collagen 1 hydrogels for a 3D invasion assay [supplementary material Fig. 2(c)], then treated with HGF after 4 days of growth to assess tubule formation. Both shCDH3-1 and shCDH3-2 cysts did not form tubules or branches in response to HGF [supplementary material Fig. 2(d)]. Furthermore, we loaded cysts into microfluidic devices to perform our CellXMD assay. Results show both shCDH3 clone cysts had no significant changes in cluster morphology (i.e., circularity and perimeter) before or after HGFtreatment [supplementary material Figs. 2(e) and 2(f)]. Matrix deformation rates were significantly smaller for shCDH3 clone cysts compared to shGFP-scramble control [supplementary material Fig. 2(g)]. These findings verify CDH3 is necessary for tubule formation, which coincides with our earlier findings with CDH3 depletion and prior work.^16^

We also quantified migration patterns, cyst morphology, and matrix deformation rates for epithelial cysts where E-cadherin (CDH1), another classical cadherin, was depleted (CDH1^-/-^). CDH1^-/-^ cysts were loaded into microfluidic devices for the CellXMD assay, as described earlier [Fig. 1(a)]. Prior to HGF stimulation, all CDH1^-/-^ cysts failed to exhibit a RCM phenotype but instead adopted a non-rotational phenotype [supplementary material Figs. 3(a)–3(c), supplementary material Video 6]. However, unlike CDH3^-/-^ cysts, upon HGF stimulation, 100% of CDH1^-/-^ cysts adopted an ICM phenotype [supplementary material Figs. 3(a)–3(c), supplementary material Video 7]. CDH1^-/-^ cysts also exhibited increased velocity upon HGF stimulation [supplementary material Fig. 3(d)], correlating to the change of migration phenotype from non-rotational to ICM. CDH1^-/-^ cysts had decreased circularity and increased perimeter with HGF simulation [supplementary material Figs. 3(e) and 3(f)], indicating a similar response to HGF as WT cysts [Figs. 2(d) and 2(e)]. The transition to the ICM phenotype correlated with an increased number of protrusions and increased ECM deformation rates compared to the non-HGF-treated group [supplementary material Figs. 3(g) and 3(h)]. Together, these findings demonstrate CDH3 is a key regulator for cysts to adopt an ICM phenotype and identify CDH1 as a potential regulator of the RCM phenotype [supplementary material Fig. 3(i)].

### D. In CDH3-depleted epithelial cysts, RhoA activation rescues the ICM phenotype associated with more vinculin focal adhesions

**FIG. 4.**
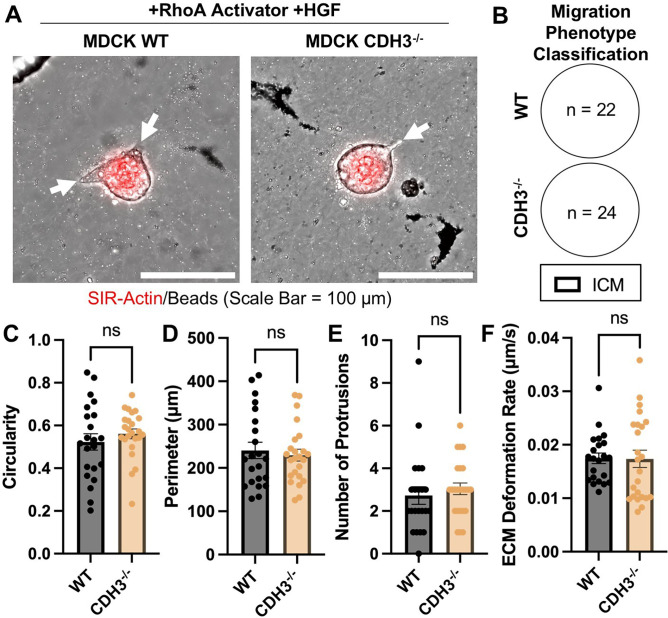
Rho activation generates equal HGF response in WT and CDH3^-/-^ cell types. (a) Representative images of MDCK epithelial cysts, WT and CDH3^-/-^, treated with RhoA activator and HGF (+Rho Activator +HGF) (white arrows highlight protrusions). (b) Classification of migration mode. (c) Circularity, (d) perimeter, (e) number of protrusions, and (f) ECM deformation quantification of WT and CDH3^-/-^ MDCK cysts treated with Rho activator and HGF. n = 22–24 cysts per condition, from at least three independent trials. Mean ± SEM, ns = not significant; Welch's t-test.

In our previously published work, we demonstrated that depletion of CDH3 results in decreased active RhoA.^16^ Thus, we asked whether RhoA affects the collective migration phenotype. We treated CDH3^-/-^ cysts with a RhoA activator and quantified changes in collective migration phenotype in response to HGF treatment. After activating RhoA, CDH3^-/-^ cysts became responsive to HGF treatment and adopted the ICM phenotype, similar to WT cysts [Figs. 4(a) and 4(b)]. Furthermore, treated CDH3^-/-^ cysts had similar circularity, perimeter, number of protrusions, and ECM deformation rate as WT cysts [Figs. 4(c)–4(f)]. Together, these findings demonstrate RhoA rescues responsiveness of CDH3^-/-^ cysts to HGF, enabling cyst protrusion formation, correlating to cyst morphology and matrix deformation rates similar to WT cysts.

**FIG. 5.**
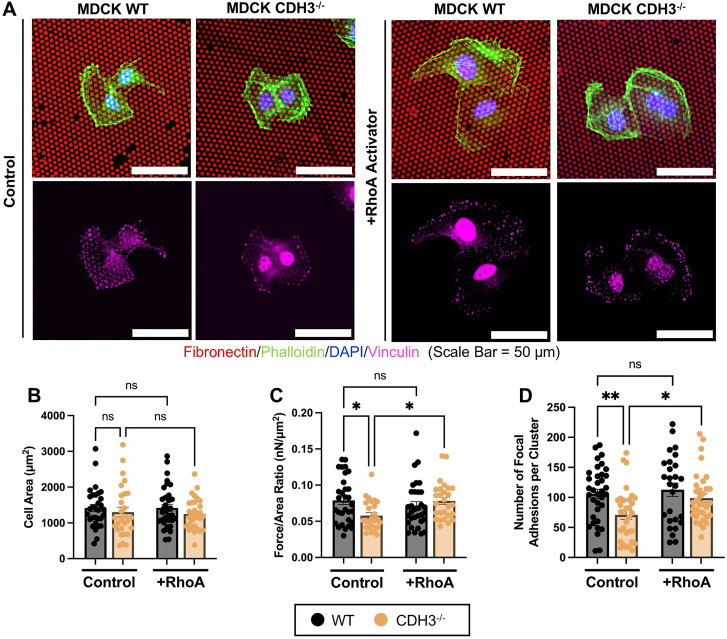
MDCK WT cells exhibit increased focal adhesions associated with increased 2D traction forces in relation to MDCK CDH3^-/-^ cells but can be rescued via RhoA activation. (a) Representative images of MDCK 2D WT and CDH3^-/-^ cell clusters, both untreated (Control) and treated with RhoA activator (+RhoA Activator). Quantification of (b) individual cell area and (c) force/area ratio. (d) Quantification of number of focal adhesions per cell cluster. n = 27–35 clusters per condition, from at least three independent trials. Mean ± SEM, ns = not significant, ^*^p < 0.05, ^**^p < 0.01; ANOVA with Tukey's multiple comparisons test.

We further investigated mechanical differences between RCM CDH3^-/-^ cysts and RhoA-activated ICM CDH3^-/-^ cysts. First, we investigated how CDH3 and RhoA regulate 2D cell–matrix forces. To do this, we used a HexForce assay^41^ and measured traction forces by quantifying deflection of fibronectin/collagen-coated dots by cell clusters, with and without RhoA activator treatment [Fig. 5(a)]. For all measurements, we focused on cell clusters (two to six cells) with similar area [Fig. 5(b)]. Findings reveal CDH3^-/-^ cell clusters exert less force per area (nN/*μ*m^2^) compared to WT clusters, and treatment with Rho activator rescues the force exertion to similar levels as WT clusters [Fig. 5(c)]. We further stained for vinculin, a focal adhesion protein, to understand whether changes in focal adhesions contribute to CDH3 and RhoA interactions. Before RhoA activation, CDH3^-/-^ clusters had significantly less focal adhesions per cluster compared to WT control [Fig. 5(d)]. After RhoA activation, CDH3^-/-^ cells had a similar number of focal adhesions per cluster as WT cells [Fig. 5(d)], thus demonstrating a link between CDH3 and vinculin, which is mediated by RhoA signaling in regulating matrix deformations associated with the ICM phenotype. To confirm the Rho/ROCK pathway mediates traction forces and focal adhesion formation, we treated WT cells with the ROCK inhibitor Y27632. After treatment with a ROCK inhibitor, clusters had decreased cell area, decreased force/area ratios, and fewer focal adhesions compared to WT non-treated cells (supplementary material Fig. 4). Finally, we stained and quantified actin stress fibers in a migrating sheet (supplementary material Fig. 5). CDH3^-/-^ cells at the leading edge of the 2D sheet exhibited a decreased number of stress fibers compared to WT cells (supplementary material Fig. 5), further suggesting CDH3-depleted cells have less actomyosin contractility, correlated with decreased traction forces and number of focal adhesions, compared to WT control cells [Figs. 5(c) and 5(d)].

## III. DISCUSSION

In this study, we investigated how epithelial cysts can adopt RCM or ICM migration phenotypes, and correlated migration phenotypes with matrix deformation rates. Our findings demonstrate epithelial cysts have a RCM phenotype and must transition to an ICM phenotype to undergo tubulogenesis. We demonstrate that ICM phenotypes are associated with higher matrix deformation rates compared to RCM phenotypes. We also demonstrate CDH3 is essential for epithelial cysts to transition from the RCM to the ICM phenotype, and CDH1 is essential for epithelial cysts to adopt the RCM phenotype. Finally, we demonstrate the ICM phenotype can be rescued in CDH3-depleted cysts with RhoA activation, mediated by increased matrix deformation rates and number of focal adhesions.

Here, we demonstrated that higher matrix deformation rates correlate with the ICM phenotype, and lower matrix deformation rates correlate with the RCM phenotype. Matrix deformation rate is an important parameter to study because it can reveal how cells interact and communicate with their environment.^42–44^ For translational work, understanding how cells adapt to their environment through changing migration behavior and mechanical factors can help create novel therapeutic opportunities.^45–47^ For example, manipulating pathways involved in adaptive migration could be a future avenue to tune immune cell recruitment for therapies. Similarly, cancer cell invasion migration could be tuned to decrease their mechanical adaptability to different tissue landscapes as a way to target metastasis.^46^ Many studies have measured mechanical factors such as 2D force exertions, cell contractility or adhesion strength, and correlated them with cell spreading or invasion.^48–50^ Other studies have probed cell forces in response to mechanical microenvironment cues, including confinement and stiffness.^44,51^ High matrix deformation rates can indicate active migration, increased matrix remodeling, and increased forces exerted on the matrix.^36,42^

To our knowledge, our study is the first to correlate the ICM phenotype with higher 3D matrix deformation rates compared to the RCM phenotype. In the literature, others have linked increased metastatic potential with single-cell traction stresses.^36^ Another group demonstrated the ICM phenotype is dependent on MMP-mediated matrix remodeling in epithelial-derived breast cancer cells.^20^ Other groups have investigated how cell division is associated with switching of collective cell rotation direction and changes in extensile forces and velocity fields in cell monolayers.^52,53^ One limitation of our experimental setup is that we cannot keep track of cysts before and after HGF treatment, so we do not know if there is a sequence for transitioning between migration phenotypes (i.e., cysts that are non-rotational must first undergo RCM before undergoing the ICM phenotype). In future work, we can add a grid pattern to our microfluidic model so that we can have a systematic manner to mark each cyst while imaging. Also, in future studies, we can quantify changes in matrix remodeling and cell division when epithelial cysts are undergoing tubulogenesis (i.e., ICM phenotype) to generate a comprehensive understanding of how each of these factors are interlinked and contribute to the collective cell migration phenotype.

In this study, we demonstrate that breast cancer clusters send out fewer protrusions when cultured in aligned collagen microfluidic devices compared to randomly oriented collagen microfluidic devices. These findings further correlate with our prior studies, as well as work by others, demonstrating aligned collagen can act as a structural, mechanical cue for cells to migrate in the direction of collagen fibers.^17,18,54,55^ In order for migration to occur, it is well established that cells generate protrusions that are long-lasting.^8,20^ Observations by others demonstrate single cells in aligned collagen send out fewer protrusions, but they are longer in length and persistent, compared to cells in randomly oriented collagen that send out short and transient protrusions.^53,54^ Our findings further extend these observations to collective breast cancer cell clusters, and we show that the protrusions generated by breast cancer clusters in aligned collagen have higher deformation rates compared to protrusions of clusters in randomly oriented collagen. One limitation of our study is that we did not correlate collective migration phenotypes (ICM vs RCM) in different matrix architectures. In future work, we will investigate how collagen architecture affects collective migration phenotypes (ICM vs RCM) and subsequent cell protrusion generation and matrix deformation rates.

Our findings contribute to our understanding of how the collective cell migration phenotype is regulated by cell–cell adhesions. Our study demonstrates epithelial cysts use CDH3 to maintain the RCM phenotype, and without it, cysts cannot transition to ICM even with HGF stimulation. These findings correlate with previous literature, which identified decreased invasion of breast tumor organoids when CDH3 was knocked down^39^ and demonstrated CDH3-depleted epithelial cysts lose their ability to form stable cell protrusions.^16,17^ Our study also demonstrated that depletion of CDH1 inhibits epithelial cysts from adopting the RCM phenotype but can still adopt the ICM phenotype after HGF stimulation. These findings suggest that CDH1 is not the primary regulator of cell protrusions and subsequent migration. In a CDH1^-/-^ *Drosophila* model, findings reveal CDH1^-/-^ cells are still motile but migrate with a slower speed and loss of directionality.^56^ E-cadherin (CDH1) mechanically couples border cells via the actomyosin cytoskeleton, and this is necessary for directional collective migration during *Drosophila* development.^57^ These findings in *Drosophila*, combined with our findings that CDH1^-/-^ prevents the RCM phenotype, further demonstrate that CDH1 regulates directionality since cells in RCM follow a circular pattern to migrate collectively.

In our prior work, we demonstrated CDH3-depleted cells have significantly less activated RhoA, and we can rescue tubulogenesis using a RhoA activator.^16^ In this study, we build on our prior work by measuring changes in matrix deformation rates upon rescuing tubulogenesis in CDH3-depleted cysts. We showed that activating RhoA in CDH3-depleted cells restored matrix deformation rates and ICM migration phenotypes, in response to HGF, to similar levels as WT cysts. Since the ICM phenotype is associated with the ability of cells to adhere to the ECM, we investigated changes in the focal adhesion protein, vinculin, as a linker used by cells to generate protrusions into the ECM. We clearly demonstrated that vinculin and CDH3 are interlinked since CDH3-depleted cells had fewer focal adhesions, and this relationship is mediated by RhoA, since the number of focal adhesions returned to wild-type levels after RhoA activation. Work by others has focused on the link between CDH1 and vinculin; one study demonstrated that when CDH1 is under tension, vinculin binds to α-catenin to stabilize the cell–cell junction.^58^ Additionally, knocking down vinculin resulted in decreased CDH1 expression^59^ and decreased traction forces.^60,61^ In addition to focal adhesions, there may be other cell surface receptors involved in regulating mechanical cell response: a prior study demonstrated RhoA activator treatment can rescue contractility of ZO-1, a tight junction protein, depleted MDCK cells, by increasing actin polymerization and traction forces in 2D collective cell sheets.^62^ Another group demonstrated that diaphanous-related formin 1 (Dia1) regulates matrix deformations and the ability to generate mature cell protrusions, but not the migration phenotype.^63^

In conclusion, we correlated RCM and ICM phenotypes with 3D matrix deformation rates in microfluidic models of epithelial tubulogenesis. Our work demonstrates epithelial cysts exist in the RCM phenotype before tubulogenesis and transition to the ICM phenotype when undergoing HGF-stimulated tubulogenesis. Findings from our study suggest a previously unappreciated mechanism by which CDH3 regulates cell migration phenotype. We show that depleting CDH3 supports the RCM phenotype and prevents epithelial cysts from adopting the ICM phenotype after HGF stimulation. CDH3-depleted RCM cysts do not generate sustained protrusions and have lower overall extracellular matrix deformation rates compared to WT cysts with the ICM phenotype. Furthermore, our studies demonstrate that the ICM phenotype can be rescued in CDH3-depleted cysts through treatment of the RhoA activator. From our 2D studies, we also suggest vinculin is a regulator of ECM deformation during ICM mediated by RhoA and CDH3. These findings enhance our understanding of developmental processes, diseased states such as fibrosis, and cancer progression.

## IV. METHODS

### A. Cell sources

All cells were cultured in DMEM 1× with 10% fetal bovine serum (FBS) and 1% penicillin–streptomycin (P/S) at 37 °C and 5% CO_2_. Epithelial cell clusters, or cysts, were generated from Madin–Darby canine kidney (MDCK) cell lines: wild-type (WT) gifted by Dr. Daniel Conway (Ohio State University), E-cadherin knockout (CDH1^-/-^) gifted by Dr. Sanjeevi Sivasankar and Dr. Soichiro Yamada (UC Davis),^64^ and P-cadherin knockout (CDH3^-/-^) developed in our laboratory.^16,17^ We used U6-gRNA/CMV-Cas9_GFP (gRNA sequence: 5′-GAGGCACCGTTCTCTGATA-3′; Sigma-Aldrich) via lipofectamine transfection of MDCK WT cells.^16^

Two shCDH3 MDCK cell lines were generated using lentiviral transduction: shCDH3-1 GFP (shRNA sequence: 5′-AAGGAAAGCACTGAAGATCTTCCCATTCG-3′; Origene), shCDH3-2 GFP (shRNA sequence: 5′-GTGGAAATTCTCGATGCTAATGACAATGC-3′; Origene), and shGFP-scramble control was used (shRNA sequence: 5′-GCACTACCAGAGCTAACTCAGATAGTACT-3′; Origene). Cells were treated with 3–5 *μ*g/ml of puromycin and seeded sparsely in 100 mm dishes, and colonies were selected using cloning rings (22877-254 and 28877-252, Corning). Western blotting was performed to confirm CDH3 knockdown and control in our selected colonies.

PyMT cell lines were obtained from previously dissociated MMTV-PyMT tumor-bearing mice [gift from Dr. Paula D. Bos (VCU)].^65,66^ Primary tumors were isolated from MMTV-PyMT mice (a gift from Dr. Gregory Longmore, Washington University) and isolated down to cell clusters known as tumor organoids using collagenase and trypsin solution [approved IACUC protocol (AD10002197)].^19,39,67^

### B. Microfluidic device fabrication

A set of microfluidic devices that can generate different matrix architectures was used. The microfluidic devices can produce an aligned matrix architecture or random fiber orientation architecture (herein referred to as “aligned” or “random,” respectively).^17^ Microfluidic devices were fabricated using standard photolithography and soft lithography techniques in the Virginia Microelectronics Center (Richmond, VA).^68^ Briefly, SU-8 2075 was spun onto silicon wafers to create a 100 *μ*m thick coating. A custom mask was placed onto the photoresist to selectively introduce UV light and cross-link the SU-8. The SU-8 was developed, and the silicon wafers with the custom device molds were silanized with Trichloro(1*H*,1*H*,2*H*,2*H*-perfluorooctyl) silane (Sigma 448931) to increase hydrophobicity. Sylgard 184 polydimethylsiloxane (PDMS) was mixed at a 1:10 ratio of curing agent to base and degassed. The PDMS was poured onto the custom molds and cured at 70 °C for a 3-h minimum. Cured PDMS was cut from the molds, and inlet and outlet holes were punched. PDMS slabs were plasma bonded (Harrick Plasma) to microscope slides (75 × 50 mm^2^) or micro cover glasses (VWR micro cover glasses, 40 × 24 mm^2^). Devices were sterilized via UV treatment (1 h) before introducing cells.

### C. Epithelial cyst generation

#### 1. Microfluidic devices

MDCK cells were seeded (7500 cells/well) with media and 2% Matrigel (Corning CB-40230) into 8-well glass-bottom chamber slides on top of a 40 *μ*l bed of Matrigel.^17,69,70^ MDCK cysts were cultured for 4–6 days to ensure the presence of a lumen and loaded into microfluidic devices as previously described.^17^ Cysts were treated with recombinant human hepatocyte growth factor (HGF; 50 ng/ml, Bio-Techne 294-HGN) to induce epithelial tubulogenesis.^15–17^

#### 2. 3D invasion assays

An underlay of rat tail collagen I (2 mg/ml, 10 *μ*l) was coated onto the bottom of 8-well chamber slides. MDCK cells (2500 cells/well) in culture media were then mixed with collagen I (4 mg/ml, rat tail collagen 1) in a 1:1 ratio to achieve a final concentration of 2 mg/ml of collagen. The hydrogels were cultured at 37 °C for 3 days, then HGF (50 ng/ml) was added daily for 4 days. On day 7, immunofluorescence staining was performed.

### D. Matrix deformation assay

Cells (PyMT single cells) or clusters (freshly isolated MMTV-PyMT primary tumor organoids or MDCK epithelial cysts) were mixed with 2 mg/ml rat tail collagen 1 (Corning) with 1% fluorescent beads (Invitrogen F8815) and loaded into microfluidic devices.^17^ Media with SIR-actin (100 nM, Cytoskeleton) and verapamil (1 nM) was added to visualize cell bodies during live-cell imaging, and samples were incubated overnight. Live-cell imaging was performed the day after loading cells into devices by taking images every 10 min for ten cycles (1 h and 40 min) at 37 °C (Nikon Ti2, 20× objective). Each cell or cluster was imaged as a z-stack with 5 *μ*m spacing in the z-direction, using brightfield (to visualize beads) and Cy5 channels (to visualize actin of cell bodies). For HGF stimulation experiments, live-cell imaging was performed again after 24 h of HGF stimulation. For RhoA activation, RhoA treatment was performed after MDCK cysts were loaded into microfluidic devices and serum starved for 24 h. RhoA activator (1 *μ*g/ml, Cytoskeleton, CN03) was added into the fluidic lines for 24 h in serum-free media (DMEM 1× + 1% penicillin–streptomycin), and then HGF (50 ng/ml) was added to fluidic lines overnight for live-cell imaging to be performed the next day.

Image processing was performed to remove background and XYZ drift (Correct 3D drift plugin) in FIJI. Image stacks were cropped to ensure each cell or cluster is centered in the XY direction: single cells (140 × 140 *μ*m^2^) and clusters (240 × 240 *μ*m^2^). For each cell and cluster, the center z-stack was selected and cropped to ensure an equal number of z-slices above and below the center slice. Brightness and contrast were adjusted to enhance the beads and cells for detection, separated into channels (brightfield and actin), and saved as TIFF files. Images are then imported into our custom MATLAB code (CellXMD), which we created using the Bio-Formats toolbox and the published track function.^71,72^ The code converts the actin channel stack to a solid binary image, overlaid on the brightfield image containing beads to minimize false detection. In our code, we calculated matrix deformation based on bead displacement around the cells (supplementary material S1, MATLAB 2023a) by first detecting the centroid [x,y, Eq. (1)] of each bead in all z-frames at each time point. Using the publicly available “track.m” function from *The MATLAB Particle Tracking Code Repository*,^72^ each bead is labeled and tracked across all time frames. For each bead in a z-frame, a net displacement is obtained across each time frame. Beads that do not appear in multiple time frames are removed from final displacement calculations. For each labeled bead, the displacement of the centroid in each time frame (t) is calculated (in micrometers), averaged, and divided by the time interval of each frame rate as follows (in seconds):

$$\text{Average}\;\text{ECM}\;\text{Deformation}\;\text{Rate}\;\text{per}\;\text{Bead}=\frac{\text{mean}(\sqrt{{{(x}_{t_{1}}-x_{t_{0}})}^{2}+{{(y}_{t_{1}}-y_{t_{0}})}^{2}})}{\text{time}\;\text{between}\;\text{frames}}.$$ (1)

To account for minute drifts in the X–Y direction during imaging, the code implements a common vector by identifying the median of all individual bead deformation rates and subtracting such value. Average ECM deformation rates per bead are then sorted into respective z-slices and averaged together to obtain a deformation rate for each z-slice and averaged all together to obtain a final average ECM deformation rate for each cyst (micrometers/second). This code utilizes the Bio-Formats toolbox for accessing images and OME-Meta data.^71^ The entire CellXMD MATLAB code is provided in supplementary material S1.

### E. 2D traction force assay

To measure 2D matrix forces, we utilized the HexForce assay.^41^ Briefly, 25 mm glass coverslips were spin coated with PDMS to form a deformable substrate (3 kPa). Negative dot grid and blank stamps were cast from 1:10 of curing agent to base PDMS. PDMS-coated coverslips were patterned with a 20:1 solution of collagen (Cultrex rat tail collagen 1) doped with rhodamine fibronectin (Cytoskeleton) to visualize the hexagonal dot grid. The MDCK cells (50 000/sample) were cultured on top of the patterned coverslips for 24 h in serum-free media (1× DMEM with 1% penicillin–streptomycin). To evaluate the effect of actomyosin contractility on traction forces and vinculin, we treated with RhoA activator (1 *μ*g/ml, Cytoskeleton, CN03) or ROCK inhibitor, Y27632 (40 *μ*M, Cayman Chemical), for 24 h. Samples were fixed in 4% paraformaldehyde for 5 min, washed in 1× PBS, and blocked in 0.1% BSA for 5 min. Cells were stained with primary antibodies tagged with fluorescence for 30 min at 37 °C and mounted onto a glass microscope slide. The phalloidin and rhodamine fibronectin images were inputted into the HexForce custom MATLAB code^41^ to quantify cell area and generate force/area ratios from dot displacement. Vinculin immunofluorescence images were used to quantify the number and size of focal adhesions using the focal adhesion analysis server (FAAS).^73^

### F. 2D cell sheet assay

Glass-bottom dishes (D35-20-1-N, Cellvis) were coated with 20 *μ*g/ml of fibronectin (F1141, Sigma-Aldrich) for 15–20 min at room temperature. After, dishes were washed twice with PBS and dried before adding 2-well culture inserts (Ibidi) to create a defined barrier. MDCK cells (49 000) were seeded into each chamber of the well insert, followed by incubation overnight for the cells to form a monolayer. The next morning, culture inserts were removed, and culture media was added to each dish. To evaluate the migration ability of cells, samples were imaged every 2 h over 8 h, and the fold change in wound area normalized to 0 h was calculated based on the change in wound area. For actin stress fiber analysis, cells were cultured for 24 h after insert removal, and then immunofluorescence staining was performed. Samples were fixed with 4% paraformaldehyde for 10 min at room temperature. After, samples were washed twice with PBS and incubated with blocking buffer (2% bovine serum albumin and 0.1% Tween20 in 1× PBS) for 1 h at 4 °C. Cells were stained with primary antibody overnight at 4 °C. The next day, samples were washed with PBST (1× PBS with 0.1% Tween20) three times, and the secondary antibody was incubated for 1 h. Samples were counterstained with DAPI. Samples were mounted with glass coverslips using Fluoromount-G™ Mounting Medium (Invitrogen) and imaged. Leading edge cells of immunofluorescence images of F-actin stress fibers were analyzed in CT-FIRE (V3.0 Beta) software^74^ using MATLAB (version 2024b). Parameters in CT-Fire (“s_xlinkbox,” “Number of selected scales,” and “Minimum fiber length [pixels]”) were adjusted for each image to optimize fiber selection. The number of fibers detected was recorded for each image.

### G. Immunofluorescence staining

#### 1. Microfluidic devices

Devices were fixed with 10% paraformaldehyde for 24 h, washed with PBST (1× PBS with 0.1% Tween20) for 24 h, and blocked (1× PBS with 0.1% Tween20 and 2% BSA) for 48 h.^17^ Primary and secondary antibodies were added for 48 h each.

#### 2. 3D invasion assay

Samples were fixed with 4% paraformaldehyde, washed with 1× PBS three times, and blocked (in Abdil) for 1 h. Primary and secondary antibodies were added for 12 h.

The antibodies used are F-actin (Alexa Fluor™ phalloidin, Invitrogen), luminal marker GP135 (Anti-Podocalyxin, Sigma-Aldrich MABS1327), vinculin (Alexa Fluor™ 647 Anti-Vinculin Antibody at 1:400×, Abcam ab196579), and species-matched secondary antibodies (Alexa Fluor™, Invitrogen). All samples were counterstained for nuclei (DAPI, Invitrogen D1306). Imaging was performed using confocal microscopy (Zeiss 980, 20×, Nanomaterials Core Characterization Facility at VCU, Richmond, VA).

### H. Analysis

#### 1. Cyst morphology

Brightfield images of cysts, containing metadata with pixel-to-micrometer conversions, were inputted into FIJI to create z-projections and perform thresholding to generate binary images. For each cyst, area and perimeter values were obtained from their respective binary image. We calculated circularity using the shape descriptors tool in FIJI as follows:

$$\text{Circularity}=(4\pi \times \text{Area})/P\text{erimete}r^{2}.$$ (2)

Protrusions were quantified by inputting binary cell or cluster images into a custom-generated MATLAB code (optimized from our original code^17^). Briefly, the MATLAB code uses the area of the cell or cluster and overlays a perfect circle with the same area and centroid over the image. Any extension, or protrusion, outside of the circular threshold was counted as a protrusion, or branch in the case of the 3D invasion assay.^16^ The code outputs a number, length, and angle of each protrusion or branch relative to the centroid (code is provided in supplementary material S2).

#### 2. Phenotype classification

Separated TIFF files were formed into videos to characterize the MDCK cyst migration phenotype. Specifically, the actin channel was used to observe whether there was luminal rotation (spinning) or not (non-spinning). Cysts with protrusions and active invasion were classified as translating.

#### 3. Migration maps and velocity

Masks of binary images of each z-projected time frame for individual cysts were generated in FIJI, then the centroid of each time frame was tracked. Upon characterization of migration phenotypes, migration maps and velocities were formed and calculated with a custom MATLAB code, using centroids and frame rate inputs.

### I. Statistical analysis

All experiments were performed at least three times independently with technical replicates. For cell deformation studies, 10–46 cells or clusters were analyzed per study. Statistical analysis was performed in GraphPad PRISM. Welch's t-test was used for comparisons between MDCK cyst morphology and matrix deformation rates with and without HGF, MDCK cysts with RhoA activator, and stress fibers of MDCK cell types. Two-way ANOVA with Tukey's *post hoc* analysis was performed for comparisons between cyst migration phenotypes with and without HGF, traction force experiments with and without RhoA activator, focal adhesions, and shCDH3 morphology. An unpaired t-test was performed for comparisons between breast tumor organoids and CDH1^-/-^ cyst morphology and matrix deformation rate. A paired t-test was performed for comparisons between traction force experiments with and without a ROCK inhibitor. All data are presented as mean ± SEM. A p-value of <0.05 was considered statistically significant.

## SUPPLEMENTARY MATERIAL

See the supplementary material for videos depicting collective migration phenotypes (non-rotational, RCM, and ICM), MATLAB codes (CellXMD and protrusion analysis), and supplementary figures referenced throughout the text. CellXMD must be run in MATLAB 2023b or newer utilizing the openly available toolbox and function, bfmatlab^71^ and track.m.^72^

## Data Availability

The data that support the findings of this study are available from the corresponding author upon reasonable request.

## References

[1] G. W. Pearson and T. Hunter, “Real-time imaging reveals that noninvasive mammary epithelial acini can contain motile cells,” J. Cell Biol. 179, 1555–1567 (2007). 10.1083/jcb.200706099 18166657 PMC2373504

[2] K. Tanner, H. Mori, R. Mroue, A. Bruni-Cardoso, and M. J. Bissell, “Coherent angular motion in the establishment of multicellular architecture of glandular tissues,” Proc. Natl. Acad. Sci. U. S. A. 109, 1973–1978 (2012). 10.1073/pnas.1119578109 22308439 PMC3277511

[3] A. J. Ewald, A. Brenot, M. Duong, B. S. Chan, and Z. Werb, “Collective epithelial migration and cell rearrangements drive mammary branching morphogenesis,” Dev. Cell 14, 570–581 (2008). 10.1016/j.devcel.2008.03.003 18410732 PMC2773823

[4] A. Aman and T. Piotrowski, “Cell migration during morphogenesis,” Dev. Biol. 341(1), 20–33 (2010). 10.1016/j.ydbio.2009.11.014 19914236

[5] G. Reig, E. Pulgar, and M. L. Concha, “Cell migration: From tissue culture to embryos,” Development 141(10), 1999–2013 (2014). 10.1242/dev.101451 24803649

[6] B. Vasiev, A. Balter, M. Chaplain, J. A. Glazier, and C. J. Weijer, “Modeling gastrulation in the chick embryo: Formation of the primitive streak,” PLoS One 5(5), e10571 (2010). 10.1371/journal.pone.0010571 20485500 PMC2868022

[7] D. Wu, K. M. Yamada, and S. Wang, “Tissue morphogenesis through dynamic cell and matrix interactions,” Annu. Rev. Cell Dev. Biol. 39, 123–144 (2023). 10.1146/annurev-cellbio-020223-031019 37315160 PMC11452922

[8] P. Friedl and D. Gilmour, “Collective cell migration in morphogenesis, regeneration and cancer,” Nat. Rev. Mol. Cell Biol. 10, 445–457 (2009). 10.1038/nrm2720 19546857

[9] A. L. Pollack, R. B. Runyan, and K. E. Mostov, “Morphogenetic mechanisms of epithelial tubulogenesis: MDCK cell polarity is transiently rearranged without loss of cell-cell contact during scatter factor/hepatocyte growth factor-induced tubulogenesis,” Dev. Biol. 204, 64–79 (1998). 10.1006/dbio.1998.9091 9851843

[10] B. Lubarsky and M. A. Krasnow, “Tube morphogenesis: Making and shaping biological tubes,” Cell 112(1), 19–28 (2003). 10.1016/S0092-8674(02)01283-7 12526790

[11] P. Friedl and R. Mayor, “Tuning collective cell migration by cell-cell junction regulation,” Cold Spring Harbor Perspect. Biol. 9, a029199 (2017). 10.1101/cshperspect.a029199 PMC5378050 28096261

[12] A. K. Mishra, J. P. Campanale, J. A. Mondo, and D. J. Montell, “Cell interactions in collective cell migration,” Development 146, dev172056 (2019). 10.1242/dev.172056 31806626 PMC7375824

[13] F. P. Hutterer, B. Buchmann, L. K. Engelbrecht, and A. R. Bausch, “Collective cell migration during human mammary gland organoid morphogenesis,” Biophys. Rev. 3, 041401 (2022). 10.1063/5.0089767 PMC10903482 38505519

[14] R. Mayor and S. Etienne-Manneville, “The front and rear of collective cell migration,” Nat. Rev. Mol. Cell Biol. 17, 97–109 (2016). 10.1038/nrm.2015.14 26726037

[15] P. Leroy and K. E. Mostov, “Slug is required for cell survival during partial epithelial-mesenchymal transition of HGF-induced tubulogenesis,” Mol. Biol. Cell 18, 1943–1952 (2007). 10.1091/mbc.e06-09-0823 17344479 PMC1855025

[16] S. K. Tran, J. Y. Lichtenberg, C. E. Leonard, J. R. Williamson, H. R. Sterling, G. K. Panek, A. H. Pearson, S. Lopez, C. A. Lemmon, D. E. Conway *et al.*, “P-cadherin-dependent adhesions are required for single lumen formation and HGF-mediated cell protrusions during epithelial morphogenesis,” iScience 28, 111844 (2025). 10.1016/j.isci.2025.111844 39981519 PMC11840494

[17] J. Y. Lichtenberg, C. E. Leonard, H. R. Sterling, V. Santos Agreda, and P. Y. Hwang, “Using microfluidics to align matrix architecture and generate chemokine gradients promotes directional branching in a model of epithelial morphogenesis,” ACS Biomater. Sci. Eng. 10, 4865–4877 (2024). 10.1021/acsbiomaterials.4c00245 39007451 PMC11322918

[18] J. Y. Lichtenberg, E. Ramamurthy, A. D. Young, T. P. Redman, C. E. Leonard, S. K. Das, P. B. Fisher, C. A. Lemmon, and P. Y. Hwang, “Leader cells mechanically respond to aligned collagen architecture to direct collective migration,” PLoS One 19, e0296153 (2024). 10.1371/journal.pone.0296153 38165954 PMC10760762

[19] P. Y. Hwang, A. Brenot, A. C. King, G. D. Longmore, and S. C. George, “Randomly distributed K14þ breast tumor cells polarize to the leading edge and guide collective migration in response to chemical and mechanical environmental cues,” Cancer Res. 79, 1899–1912 (2019). 10.1158/0008-5472.CAN-18-2828 30862718 PMC6467777

[20] S. K. Ranamukhaarachchi, A. Walker, M.-H. Tang, W. D. Leineweber, S. Lam, W.-J. Rappel, and S. I. Fraley, “Global versus local matrix remodeling drives rotational versus invasive collective migration of epithelial cells,” Dev. Cell 60, 871 (2025). 10.1016/j.devcel.2024.11.021 39706188 PMC11945606

[21] A. Glentis, C. Blanch-Mercader, L. Balasubramaniam, T. Beng Saw, S. Janel, A. Douanier, B. Delaval, F. Lafont, C. Teck Lim, D. Delacour *et al.*, “The emergence of spontaneous coordinated epithelial rotation on cylindrical curved surfaces,” Sci. Adv. 8, 5406 (2022). 10.1126/sciadv.abn5406 PMC9473582 36103541

[22] A. S. Chin, K. E. Worley, P. Ray, G. Kaur, J. Fan, and L. Q. Wan, “Epithelial cell chirality revealed by three-dimensional spontaneous rotation,” Proc. Natl. Acad. Sci. U. S. A. 115, 12188–12193 (2018). 10.1073/pnas.1805932115 30429314 PMC6275504

[23] K. Doxzen, S. R. Vedula, M. C. Leong, H. Hirata, N. S. Gov, A. J. Kabla, B. Ladoux, and C. T. Lim, “Guidance of collective cell migration by substrate geometry,” Integr. Biol. 5(8), 1026–1035 (2013). 10.1039/c3ib40054a 23784144

[24] M. Deforet, V. Hakim, H. G. Yevick, G. Duclos, and P. Silberzan, “Emergence of collective modes and tri-dimensional structures from epithelial confinement,” Nat. Commun. 5(1), 3747 (2014). 10.1038/ncomms4747 24796352

[25] S. R. Vedula, M. C. Leong, T. L. Lai, P. Hersen, A. J. Kabla, C. T. Lim, and B. Ladoux, “Emerging modes of collective cell migration induced by geometrical constraints,” Proc. Natl. Acad. Sci. U. S. A. 109(32), 12974–12979 (2012). 10.1073/pnas.1119313109 22814373 PMC3420172

[26] F. Ascione, S. Caserta, S. Esposito, V. R. Villella, L. Maiuri, M. R. Nejad, A. Doostmohammadi, J. M. Yeomans, and S. Guido, “Collective rotational motion of freely expanding T84 epithelial cell colonies,” J. R. Soc. Interface 20(199), 20220719 (2023). 10.1098/rsif.2022.0719 36872917 PMC9943890

[27] P. Pandya, J. L. Orgaz, and V. Sanz-Moreno, “Actomyosin contractility and collective migration: May the force be with you,” Curr. Opin. Cell Biol. 48, 87–96 (2017). 10.1016/j.ceb.2017.06.006 28715714 PMC6137077

[28] J. R. Lange and B. Fabry, “Cell and tissue mechanics in cell migration,” Exp. Cell Res. 319(16), 2418–2423 (2013). 10.1016/j.yexcr.2013.04.023 23664834 PMC3773016

[29] L. Yao and Y. Li, “Effective force generation during mammalian cell migration under different molecular and physical mechanisms,” Front. Cell Dev. Biol. 10, 903234 (2022). 10.3389/fcell.2022.903234 35663404 PMC9160717

[30] D. N. Clarke and A. C. Martin, “Actin-based force generation and cell adhesion in tissue morphogenesis,” Curr. Biol. 31(10), R667–R680 (2021). 10.1016/j.cub.2021.03.031 34033797 PMC8276969

[31] I. C. Fortunato and R. Sunyer, “The forces behind directed cell migration,” Biophysica 2(4), 548–563 (2022). 10.3390/biophysica2040046

[32] J. Stock and A. Pauli, “Self-organized cell migration across scales – From single cell movement to tissue formation,” Development 148(7), dev191767 (2021). 10.1242/dev.191767 33824176

[33] X. Trepat, M. R. Wasserman, T. E. Angelini, E. Millet, D. A. Weitz, J. P. Butler, and J. J. Fredberg, “Physical forces during collective cell migration,” Nat. Phys. 5(6), 426–430 (2009). 10.1038/nphys1269

[34] A. Bajpai, J. Tong, W. Qian, Y. Peng, and W. Chen, “The interplay between cell-cell and cell-matrix forces regulates cell migration dynamics,” Biophys. J. 117, 1795–1804 (2019). 10.1016/j.bpj.2019.10.015 31706566 PMC7031787

[35] M. Reffay, M. C. Parrini, O. Cochet-Escartin, B. Ladoux, A. Buguin, S. Coscoy, F. Amblard, J. Camonis, and P. Silberzan, “Interplay of RhoA and mechanical forces in collective cell migration driven by leader cells,” Nat. Cell Biol. 16(3), 217–223 (2014). 10.1038/ncb2917 24561621

[36] C. M. Kraning-Rush, J. P. Califano, and C. A. Reinhart-King, “Cellular traction stresses increase with increasing metastatic potential,” PLoS One 7, e32572 (2012). 10.1371/journal.pone.0032572 22389710 PMC3289668

[37] T. Koorman, K. A. Jansen, A. Khalil, P. D. Haughton, D. Visser, M. A. K. Rätze, W. E. Haakma, G. Sakalauskaitè, P. J. van Diest, J. de Rooij *et al.*, “Spatial collagen stiffening promotes collective breast cancer cell invasion by reinforcing extracellular matrix alignment,” Oncogene 41, 2458–2469 (2022). 10.1038/s41388-022-02258-1 35292774 PMC9033577

[38] R. Montesano, K. Matsumoto, T. Nakamura, and L. Orci, “Identification of a fibroblast-derived epithelial morphogen as hepatocyte growth factor,” Cell 67, 901–908 (1991). 10.1016/0092-8674(91)90363-4 1835669

[39] P. Y. Hwang, J. Mathur, Y. Cao, J. Almeida, J. Ye, V. Morikis, D. Cornish, M. Clarke, S. A. Stewart, A. Pathak *et al.*, “A Cdh3-β-catenin-laminin signaling axis in a subset of breast tumor leader cells control leader cell polarization and directional collective migration,” Dev. Cell 58, 34–50 (2023). 10.1016/j.devcel.2022.12.005 36626870 PMC10010282

[40] H. Wang, S. Lacoche, L. Huang, B. Xue, and S. K. Muthuswamy, “Rotational motion during three-dimensional morphogenesis of mammary epithelial acini relates to laminin matrix assembly,” Proc. Natl. Acad. Sci. U. S. A. 110, 163–168 (2013). 10.1073/pnas.1201141110 23248267 PMC3538193

[41] B. P. Griffin, C. J. Largaespada, N. A. Rinaldi, and C. A. Lemmon, “A novel method for quantifying traction forces on hexagonal micropatterned protein features on deformable poly-dimethyl siloxane sheets,” MethodsX 6, 1343–1352 (2019). 10.1016/j.mex.2019.05.011 31417850 PMC6690417

[42] N. Gjorevski, A. S. Piotrowski, V. D. Varner, and C. M. Nelson, “Dynamic tensile forces drive collective cell migration through three-dimensional extracellular matrices,” Sci. Rep. 5, 11458 (2015). 10.1038/SREP11458 26165921 PMC4499882

[43] M. K. Sewell-Loftin, S. V. H. Bayer, E. Crist, T. Hughes, S. M. Joison, G. D. Longmore, and S. C. George, “Cancer-associated fibroblasts support vascular growth through mechanical force,” Sci. Rep. 7, 12574 (2017). 10.1038/s41598-017-13006-x 28974764 PMC5626692

[44] J. Chen, D. Yan, and Y. Chen, “Understanding the driving force for cell migration plasticity,” Biophys. J. 122(18), 3570–3576 (2023). 10.1016/j.bpj.2023.04.008 37041746 PMC10541478

[45] X. Di, X. Gao, L. Peng, J. Ai, X. Jin, S. Qi, H. Li, K. Wang, and D. Luo, “Cellular mechanotransduction in health and diseases: From molecular mechanism to therapeutic targets,” Signal Transduction Targeted Ther. 8(1), 282 (2023). 10.1038/s41392-023-01501-9 PMC10387486 37518181

[46] H. Zhou, M. Wang, Y. Zhang, Q. Su, Z. Xie, X. Chen, R. Yan, P. Li, T. Li, X. Qin *et al.*, “Functions and clinical significance of mechanical tumor microenvironment: Cancer cell sensing, mechanobiology and metastasis,” Cancer Commun. 42(5), 374–400 (2022). 10.1002/cac2.12294 PMC9118059 35470988

[47] M. Li, N. Xi, Y.-C. Wang, and L.-Q. Liu, “Atomic force microscopy for revealing micro/nanoscale mechanics in tumor metastasis: From single cells to microenvironmental cues,” Acta Pharmacol. Sin. 42(3), 323–339 (2021). 10.1038/s41401-020-0494-3 32807839 PMC8027022

[48] S. Gupta, K. Duszyc, S. Verma, S. Budnar, X. Liang, G. A. Gomez, P. Marcq, I. Noordstra, and A. S. Yap, “Enhanced RhoA signalling stabilizes E-cadherin in migrating epithelial monolayers,” J. Cell Sci. 134(17), jcs258767 (2021). 10.1242/jcs.258767 34368835

[49] B. A. Nerger, M. J. Siedlik, and C. M. Nelson, “Microfabricated tissues for investigating traction forces involved in cell migration and tissue morphogenesis,” Cell. Mol. Life Sci. 74(10), 1819–1834 (2017). 10.1007/s00018-016-2439-z 28008471 PMC5391279

[50] D. N. Metsiou, D. Deligianni, E. Giannopoulou, H. Kalofonos, A. Koutras, and G. Athanassiou, “Adhesion strength and anti-tumor agents regulate vinculin of breast cancer cells,” Front. Oncol. 12, 811508 (2022). 10.3389/fonc.2022.811508 36052248 PMC9424896

[51] P. S. Raman, C. D. Paul, K. M. Stroka, and K. Konstantopoulos, “Probing cell traction forces in confined microenvironments,” Lab Chip 13(23), 4599–4607 (2013). 10.1039/c3lc50802a 24100608 PMC5409513

[52] M. J. Siedlik, S. Manivannan, I. G. Kevrekidis, and C. M. Nelson, “Cell division induces and switches coherent angular motion within bounded cellular collectives,” Biophys. J. 112(11), 2419–2427 (2017). 10.1016/j.bpj.2017.05.001 28591614 PMC5474845

[53] A. Doostmohammadi, S. P. Thampi, T. B. Saw, C. T. Lim, B. Ladoux, and J. M. Yeomans, “Celebrating Soft Matter's 10th Anniversary: Cell division: A source of active stress in cellular monolayers,” Soft Matter 11(37), 7328–7336 (2015). 10.1039/c5sm01382h 26265162

[54] S. P. Carey, Z. E. Goldblatt, K. E. Martin, B. Romero, R. M. Williams, and C. A. Reinhart-King, “Local extracellular matrix alignment directs cellular protrusion dynamics and migration through Rac1 and FAK,” Integr. Biol. 8(8), 821–835 (2016). 10.1039/c6ib00030d PMC4980151 27384462

[55] K. M. Riching, B. L. Cox, M. R. Salick, C. Pehlke, A. S. Riching, S. M. Ponik, B. R. Bass, W. C. Crone, Y. Jiang, A. M. Weaver *et al.*, “3D collagen alignment limits protrusions to enhance breast cancer cell persistence,” Biophys. J. 107(11), 2546–2558 (2014). 10.1016/j.bpj.2014.10.035 25468334 PMC4255204

[56] D. Cai, S.-C. Chen, M. Prasad, L. He, X. Wang, V. Choesmel-Cadamuro, J. K. Sawyer, G. Danuser, and D. J. Montell, “Mechanical feedback through E-cadherin promotes direction sensing during collective cell migration,” Cell 157(5), 1146–1159 (2014). 10.1016/j.cell.2014.03.045 24855950 PMC4118667

[57] C. L. Messer and J. A. McDonald, “Expect the unexpected: Conventional and unconventional roles for cadherins in collective cell migration,” Biochem. Soc. Trans. 51(4), 1495–1504 (2023). 10.1042/bst20221202 37387360

[58] S. Yonemura, Y. Wada, T. Watanabe, A. Nagafuchi, and M. Shibata, “α-Catenin as a tension transducer that induces adherens junction development,” Nat. Cell Biol. 12, 533–542 (2010). 10.1038/ncb2055 20453849

[59] X. Peng, L. E. Cuff, C. D. Lawton, and K. A. DeMali, “Vinculin regulates cell-surface E-cadherin expression by binding to β-catenin,” J. Cell Sci. 123, 567–577 (2010). 10.1242/jcs.056432 20086044 PMC2818194

[60] A. Rahman, S. P. Carey, C. M. Kraning-Rush, Z. E. Goldblatt, F. Bordeleau, M. C. Lampi, D. Y. Lin, A. J. García, and C. A. Reinhart-King, “Vinculin regulates directionality and cell polarity in two-And three-dimensional matrix and three-dimensional microtrack migration,” Mol. Biol. Cell 27, 1431–1441 (2016). 10.1091/mbc.E15-06-0432 26960796 PMC4850031

[61] C. T. Mierke, P. Kollmannsberger, D. P. Zitterbart, G. Diez, T. M. Koch, S. Marg, W. H. Ziegler, W. H. Goldmann, and B. Fabry, “Vinculin facilitates cell invasion into three-dimensional collagen matrices,” J. Biol. Chem. 285, 13121–13130 (2010). 10.1074/jbc.M109.087171 20181946 PMC2857131

[62] M. Jipp, B. D. Wagner, L. Egbringhoff, A. Teichmann, A. Rübeling, P. Nieschwitz, A. Honigmann, A. Chizhik, T. A. Oswald, and A. Janshoff, “Cell-substrate distance fluctuations of confluent cells enable fast and coherent collective migration,” Cell Rep. 43, 114553 (2024). 10.1016/j.celrep.2024.114553 39150846

[63] T. B. Fessenden, Y. Beckham, M. Perez-Neut, G. R. S. Juan, A. H. Chourasia, K. F. Macleod, P. W. Oakes, and M. L. Gardel, “Dia1-dependent adhesions are required by epithelial tissues to initiate invasion,” J. Cell Biol. 217, 1485–1502 (2018). 10.1083/jcb.201703145 29437785 PMC5881494

[64] R. Koirala, A. Vae Priest, C.-F. Yen, J. S. Cheah, W.-J. Pannekoek, M. Gloerich, S. Yamada, and S. Sivasankar, “Inside-out regulation of E-cadherin conformation and adhesion,” Proc. Natl. Acad. Sci. U. S. A. 118, 2021 (2021). 10.1073/pnas.2104090118/-/DCSupplemental PMC8325368 34301871

[65] N. M. Clark, L. M. Martinez, S. Murdock, J. T. deLigio, A. L. Olex, C. Effi, M. G. Dozmorov, and P. D. Bos, “Regulatory T cells support breast cancer progression by opposing IFN-γ-dependent functional reprogramming of myeloid cells,” Cell Rep. 33, 108482 (2020). 10.1016/j.celrep.2020.108482 33296659 PMC7811278

[66] P. D. Bos, G. Plitas, D. Rudra, S. Y. Lee, and A. Y. Rudensky, “Transient regulatory T cell ablation deters oncogene-driven breast cancer and enhances radiotherapy,” J. Exp. Med. 210, 2435–2466 (2013). 10.1084/jem.20130762 24127486 PMC3804934

[67] V. Padmanaban, E. M. Grasset, N. M. Neumann, A. K. Fraser, E. Henriet, W. Matsui, P. T. Tran, K. J. Cheung, D. Georgess, and A. J. Ewald, “Organotypic culture assays for murine and human primary and metastatic-site tumors,” Nat. Protoc. 15, 2413–2442 (2020). 10.1038/s41596-020-0335-3 32690957 PMC8202162

[68] D. Huh, H. J. Kim, J. P. Fraser, D. E. Shea, M. Khan, A. Bahinski, G. A. Hamilton, and D. E. Ingber, “Microfabrication of human organs-on-chips,” Nat. Protoc. 8, 2135–2157 (2013). 10.1038/nprot.2013.137 24113786

[69] J. Debnath, S. K. Muthuswamy, and J. S. Brugge, “Morphogenesis and oncogenesis of MCF-10A mammary epithelial acini grown in three-dimensional basement membrane cultures,” Methods 30, 256–268 (2003). 10.1016/S1046-2023(03)00032-X 12798140

[70] V. Narayanan, L. E. Schappell, C. R. Mayer, K. N. Dahl, J. P. Gleghorn, and D. E. Conway, “osmotic gradients in epithelial acini increase mechanical tension across E-cadherin, drive morphogenesis, and maintain homeostasis,” Curr. Biol. 30, 624–633 (2020). 10.1016/j.cub.2019.12.025 31983640 PMC7153951

[71] M. Linkert, C. T. Rueden, C. Allan, J. M. Burel, W. Moore, A. Patterson, B. Loranger, J. Moore, C. Neves, D. MacDonald *et al.*, “Metadata matters: Access to image data in the real world,” J. Cell Biol. 189, 777–782 (2010). 10.1083/jcb.201004104 20513764 PMC2878938

[72] D. Blair and E. Dufresne, see https://site.physics.georgetown.edu/matlab/code.html for “The Matlab Particle Tracking Code Repository” (2007).

[73] M. E. Berginski and S. M. Gomez, “The Focal Adhesion Analysis Server: A web tool for analyzing focal adhesion dynamics,” F1000Research 2, 68 (2013). 10.12688/f1000research.2-68.v1 24358855 PMC3752736

[74] Y. Liu, A. Keikhosravi, G. S. Mehta, C. R. Drifka, and K. W. Eliceiri, “Methods for quantifying fibrillar collagen alignment,” Methods Mol. Biol. 1627, 429 (2017). 10.1007/978-1-4939-7113-8_28 28836218 PMC6343484

